# Effectiveness of interventions to provide culturally appropriate maternity care in increasing uptake of skilled maternity care: a systematic review

**DOI:** 10.1093/heapol/czw065

**Published:** 2016-05-17

**Authors:** Ernestina Coast, Eleri Jones, Samantha R Lattof, Anayda Portela

**Affiliations:** Department of Social Policy, London School of Economics and Political Science, London WC2A 2AE, UK

**Keywords:** Antenatal care, birth, culture, maternity care, maternal health, pregnancy, systematic review, utilization

## Abstract

Addressing cultural factors that affect uptake of skilled maternity care is recognized as an important step in improving maternal and newborn health. This article describes a systematic review to examine the evidence available on the effects of interventions to provide culturally appropriate maternity care on the use of skilled maternity care during pregnancy, for birth or in the postpartum period. Items published in English, French and/or Spanish between 1 January 1990 and 31 March 2014 were considered. Fifteen studies describing a range of interventions met the inclusion criteria. Data were extracted on population and intervention characteristics; study design; definitions and data for relevant outcomes; and the contexts and conditions in which interventions occurred. Because most of the included studies focus on antenatal care outcomes, evidence of impact is particularly limited for care seeking for birth and after birth. Evidence in this review is clustered within a small number of countries, and evidence from low- and middle-income countries is notably lacking. Interventions largely had positive effects on uptake of skilled maternity care. Cultural factors are often not the sole factor affecting populations’ use of maternity care services. Broader social, economic, geographical and political factors interacted with cultural factors to affect targeted populations’ access to services in included studies. Programmes and policies should seek to establish an enabling environment and support respectful dialogue with communities to improve use of skilled maternity care. Whilst issues of culture are being recognized by programmes and researchers as being important, interventions that explicitly incorporate issues of culture are rarely evaluated.

Key Messages

- Interventions to provide culturally appropriate maternity care are primarily focused on antenatal care and have largely positive effects on uptake of skilled maternity care.
- Evidence on culturally appropriate maternity care from low- and middle- income countries is very limited.
- Research to evaluate culturally appropriate maternity care should use robust study designs and standardize the descriptions and outcome measurements of interventions.

## Introduction

Increasing the use of skilled maternity care services was a key objective under the Millennium Development Goals and continues to be a focus of the post-2015 development agenda (United Nations 2008, 2014; Langer *et al.* 2013). Minority groups across world regions frequently have poorer maternal and newborn health outcomes and may be less likely than other populations to use skilled maternity care services (Wasserman *et al.* 2007; Harris *et al.* 2010; Kildea and Van Wagner 2012). Studies have shown that cultural factors can affect uptake of maternity care services, and addressing them has been recognized as an important step in increasing the use of services (Thaddeus and Maine 1994; WHO 2003; UNFPA 2005; Camacho *et al.* 2006; Gabrysch and Campbell 2009; Castro 2012; Bohren *et al.* 2014).

There may be differences between the culture of maternity care services and the cultural practices and preferences of women and communities, in regards to childbirth settings, practices, attitudes towards illness and health, materials and/or language, for example. Perceived cultural insensitivity or poor intercultural competencies of professionals can also lead to discrimination of certain users by providers, resulting in a lack of trust in services and service providers (Gabrysch *et al.* 2009). Cultural beliefs and practices are often framed as a ‘barrier’ to the uptake of maternity care services, rather than a population characteristic which health systems need to consider in order to be responsive to communities’ needs. Providing care that takes people’s cultural preferences into account is an important component of quality of care (Tuncalp *et al.* 2015). The need for ‘culturally appropriate’ maternity care services is core to the World Health Organization’s (WHO) strategy for improving maternal and newborn health (WHO 2003) and ending preventable maternal mortality (WHO 2015a).

To provide programmes and materials that are culturally appropriate, health service managers must be able to identify and describe cultures and/or subcultures, understand their health behaviours, and apply this knowledge in planning and development activities (Kreuter *et al.* 2003). In practice, however, culture is more often assumed than assessed (Kreuter *et al.* 2003), and culture is frequently used as a synonym for ‘traditional’ or ‘backward’ as opposed to ‘modern’ by researchers, service providers and policymakers. A common approach in health interventions and their evaluations is to reduce culture to a static set of features. It is often the explicit and manifest components of culture (e.g. language, traditional dress, music) that are used as markers of culture (Fisher *et al.* 2007), the dangers of cultural stereotyping notwithstanding. Other dimensions such as shared values and assumptions may be more effective, yet much harder to incorporate. Such an approach is out of step with perspectives from contemporary anthropology in particular, and social science more generally, in which culture is understood as an outcome of multiple and on-going interactions within and between communities, their networks and their environment for an individual over their life course (Kirmayer 2012).

The WHO Department of Maternal, Newborn, Child and Adolescent Health has assessed evidence on the effectiveness of a range of interventions in improving use of skilled care for birth (WHO 2015b). Interventions to address cultural factors are one focus area. In this article, we report on a systematic review to examine evidence on the effects of interventions to provide culturally appropriate maternity care services on the uptake of skilled care during pregnancy, for birth or in the postpartum period. Skilled maternity care is defined here as the care provided by an accredited health professional (e.g. doctor, midwife or nurse) who has been educated and ‘trained to proficiency in the skills needed to manage normal (uncomplicated) pregnancies, childbirth and the immediate postnatal period, and in the identification, management and referral of complications in women and newborns’ (WHO 2004). This definition excludes traditional birth attendants (TBAs), although countries are encouraged to work with TBAs to define new roles for them, and ensure good working relations between TBAs, skilled attendants and staff in referral facilities.

A previous systematic mapping of the literature describes interventions to address cultural factors affecting women’s use of skilled maternity care (Coast *et al.* 2014). This mapping included 96 items from 35 countries across all world regions. It describes the scope of existing literature, including both the types and range of interventions and the types of documentation available, but does not report on intervention effectiveness (Coast *et al.* 2014).

In this systematic, review we focus on interventions in which providing culturally appropriate care was a primary aim, and we assess the effectiveness of these interventions for the uptake of skilled maternity care. Improving understanding of how culture can be incorporated into generic quality improvement interventions is important (Fisher *et al.* 2007). However, including such broad interventions would have prevented us from examining specific effects of providing culturally appropriate care. As far as the research team is aware, this systematic review is the first to address this research question. Other review articles that address-related questions informed the present review as relevant background literature (Anderson *et al.* 2003; Wasserman *et al.* 2007; Kildea and Van Wagner 2012; Jongen *et al.* 2014).

## Materials and methods

### Inclusion and exclusion criteria

This review focuses on studies examining the impact of interventions to provide culturally appropriate maternity care on women’s use of skilled maternity care before, during and after birth. Studies on any population in any world region were considered. Peer-reviewed journal articles and (non-peer-reviewed) grey literature, including reports, books or book chapters, whether available in print or online, were eligible for inclusion. Items published in English, French and/or Spanish between 1 January 1990 and 31 March 2014 were considered.

For inclusion, studies must have measured the impact of an implemented intervention to provide culturally appropriate maternity care for ethno-linguistic or religious groups. Only items that describe an implemented intervention are included. Items that only identified cultural factors affecting the use of skilled maternal care, and items describing a recommended (but thus far non-implemented) intervention are excluded. A cultural group is defined broadly to include any form of group or social stratum that is (considered to be) marked by its own distinctive cultural characteristics (Helman 2000; Kreuter *et al.* 2003). A non-exhaustive list of sub-populations that may be marked by their own distinctive cultural characteristics include ethnic groups, groups defined by religion, language groups, indigenous groups, tribal communities and caste-based groups. Providing culturally appropriate care must have been a primary aim of the intervention. To be included, an intervention had to be explicitly designed to accommodate a cultural group’s shared norms, values and/or beliefs, behavioural customs, and/or spoken language/s. Interventions whose primary aims or strategies are not concerned with accommodating or addressing cultural factors are excluded (e.g. interventions that exclusively address economic or geographical access barriers for a defined cultural group). Generic interventions that consider and/or accommodate cultural factors explicitly or implicitly, but not as an explicit aim or strategy, are excluded. Complex packages of interventions to provide culturally appropriate care were eligible for inclusion. Studies must have reported empirical data on an assessment of the outcome of an intervention and compared this with the outcome in a group that received no intervention or a different intervention. Studies comparing the same population before and after the intervention were considered only where data were collected for the same population at different time points.

The outcome of interest was uptake of skilled maternity care during pregnancy, for birth or in the postpartum period. Studies must have measured at least one of these outcomes:

- Birth with a skilled attendant
- Birth in a health facility
- Care with a skilled attendant or at a facility in case of maternal complications or illness
- Use of antenatal care (ANC) (with a skilled attendant)
- Timing of first ANC visit
- Postpartum care visit (with a skilled attendant)

### Searching and screening strategy

A broad precursory systematic mapping (Coast *et al.* 2014) formed the first stage of the searching and screening for this systematic review. The search strategy involved systematic searches of ten electronic databases and two targeted websites. The search terms and their combinations (Table 1) were adapted to the particulars (e.g. wildcards, truncations, capacity for complex searches, Medical Subject Headings facility) of each electronic database. The results were combined with references suggested by experts. This search generated a total of 33 227 items for screening in the first mapping phase. Items identified in the search were then screened for inclusion in the mapping, initially on the basis of title and abstract. The team screened the full text when inclusion or exclusion could not be determined on the basis of title and abstract. Quality assurance measures were incorporated into the screening process, as the process involved multiple team members. Prior to single-screening items generated by the search, the entire team independently screened the first 100 items, compared results and resolved any differences in understanding the inclusion/exclusion criteria. Any full text item that was considered problematic or borderline was double-screened, following which the team compared results and resolved any differences, with decisions made in favour of an inclusive approach where questions remained. A total of 96 items were included in the mapping.

**Table 1.** Search terms and their combinations

1. Intervention/study type terms	2. Access/use terms	3. Care terms	4. Population terms	5. Culture terms

Arrangement*	Accept*	Advice	***Antepartum terms***	Attitud*
Evaluat*	Access	ANC	Ante*natal	Behaviour*
Initiative*	Appointment*	‘Birth attendan*’	Ante*partum	Behavior*
Intervention*	Attend*	Care	Expect*^a^	Belief*
Model*	Availab*	Doctor*	Pregnan*	Believ*
Package*	Obtain	Centre	Prenatal	Caste*
Pilot*	Outreach	Center	Trimester	Communit*
Program*	Recei*	Clinic*	***Intrapartum terms***	Culture*
Project*	Seek*	Counsel*	Birth*	Cultural
Provision*	Uptake	Department*	Child*birth	Custom*
Regime*	Use	Facilit*	Intra*partum	Ethnic*
Scheme*	Utilization	Healthcare	Maternity	Indigen*
Strateg*	Utilization	Health care	Obstetric*	Language*
Trial*	Visit*	‘Health system’	Parturition	Minorit*
		Hospital*	Partus	Norm*
		Institution*	Peri*natal	Race*
		Midwif*	Deliver*^a^	Racial*
		Nurs*	Labour^a^	Religio*
		Physician*	Labor^a^	Ritual*
		PNC	***Postpartum terms***	Sub*cultur*
		Service*	Maternal	Sub*population*
		Treatment*	‘New mother’	Tradition*
		Unit*	Post*natal	Tribal*
			Post*partum	Tribe*
			PPC	Value*
			Puerper*	Participatory

^a^These terms have multiple meanings. Due to their presence in a column that narrowed a search that was otherwise very broad, they were included only in searches where their inclusion did not yield an unfeasible number of irrelevant references.

**Figure 1.**
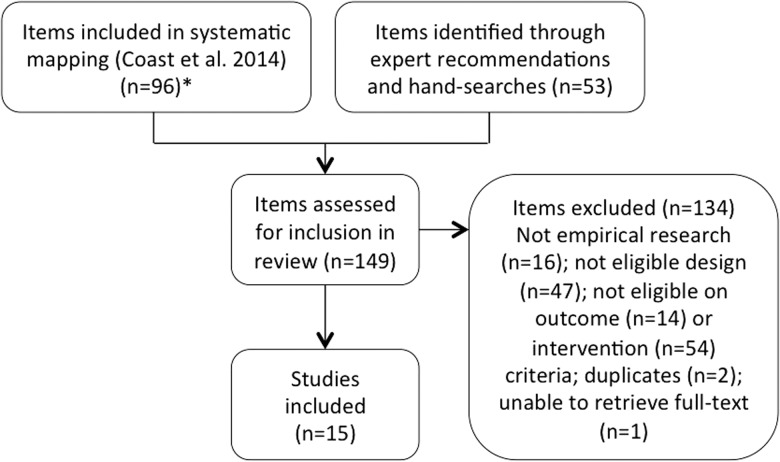
Systematic review searching and screening.

At the systematic review stage we sought additional items through hand-searches of the reference lists of included studies and relevant reviews, as well as further items suggested by experts. As much of the intervention activity related to cultural interventions, particularly in low- and middle-income countries (LMICs), is not (yet) peer-reviewed or published, expert suggestions were an important addition to the search for the systematic review. Searches and suggestions were further supplemented with items identified as potentially relevant from a separate systematic mapping of maternal health intervention studies conducted in LMICs between 2000 and 2012, described elsewhere (MASCOT/MH-SAR 2013; MASCOT Study Group 2014). Items identified through expert recommendations, hand-searches and this separate systematic mapping of maternal health intervention studies yielded a further 53 items, generating a total of 149 items for screening for the systematic review. E.J. or S.L. determined eligibility of all items, and unclear items were discussed using a protocol that is available, upon request, from the lead author. When necessary, differences were resolved by discussion with E.C. and A.P. See Figure 1 for a flow diagram of the searching and screening strategy.

### Data extraction, analysis and quality assessment

S.L. or E.J. extracted data from each included study. Data were extracted on population and intervention characteristics; study design; and definitions and data for relevant outcomes. Included studies were quality assessed in duplicate by S.L., E.C. and/or E.J. using the Effective Public Health Practice Project (EPHPP) quality assessment tool for quantitative studies (EPHPP 2010). Conducting a meta-analysis was not considered appropriate in view of the heterogeneity of interventions, populations and outcome measures within the evidence base. We present the results in a narrative format, accompanied by tables of included studies.

## Results

Fifteen papers met our inclusion criteria, evaluating the impact of 14 different interventions. All studies but two were conducted in high-income, OECD-member countries: Australia (*n* = 6) (Nel and Pashen 2003; Jan *et al.* 2004; NSW Health 2005; Panaretto *et al.* 2005, 2007; Kildea *et al.* 2012), the United States of America (USA) (*n* = 4) (Julnes *et al.* 1994; Thompson *et al.* 1998; Jewell and Russell 2000; Marsiglia *et al.* 2010), the United Kingdom (UK) (*n* = 2) (Mason 1990; Parsons and Day 1992) and Israel (*n* = 1) (Bilenko *et al.* 2007). The two exceptions were conducted in Peru (McQuestion and Velasquez 2006; Gabrysch *et al.* 2009).

All studies from Australia targeted Indigenous, Aboriginal and Torres Islander Strait women (Nel and Pashen 2003; Jan *et al.* 2004; NSW Health 2005; Panaretto *et al.* 2005, 2007; Kildea *et al.* 2012). Indigenous women were also the focus in one of the studies from Peru (Gabrysch *et al.* 2009), and the study in Israel targeted semi-nomadic Bedouin women (Bilenko *et al.* 2007). In contrast, studies from the USA and UK largely targeted migrant, ethnic minority groups, particularly Latina or Hispanic women in the USA (Julnes *et al.* 1994; Thompson *et al.* 1998; Jewell and Russell 2000; Marsiglia *et al.* 2010) and Asian women in the UK (Mason 1990; Parsons and Day 1992).

### Interventions

The papers summarize a diverse range of interventions designed to provide culturally appropriate maternity care services (see Table 2). Strategies included: using health care providers with shared cultural and/or linguistic background with service users (Thompson *et al.* 1998; Jewell and Russell 2000; Nel and Pashen 2003; Panaretto *et al.* 2005, 2007; Bilenko *et al.* 2007); providing cultural brokers, mediators or interpreters (Mason 1990; Parsons and Day 1992; Julnes *et al.* 1994; Marsiglia *et al.* 2010; Kildea *et al.* 2012); training staff to improve cultural awareness (Jan *et al.* 2004; NSW Health 2005); incorporating culturally appropriate practices (McQuestion and Velasquez 2006) and adapting the setting within which the service is provided (Gabrysch *et al.* 2009).

**Table 2.** Intervention and study details

Study	Setting and target population	Intervention or service adaptation description	Study design and sample	Effects on care utilization outcomes	Quality	Commentary
Positive effect on care utilization						
Bilenko et al. 2007	Negev desert, Israel Semi-nomadic Bedouin Arab women	A new maternal and child health clinic was established in a desert area to serve Bedouin extended families living in tribal units. The primary practitioner was an Arabic-speaking Bedouin public health nurse. A local female Bedouin liaison worker was employed.	Retrospective record review of ANC utilization by women in two successive pregnancies, one before and one after the clinic opened. The sample comprised all eligible women who registered newborns for care at the clinic in the 2-year period after it opened (*n* = 85). The same women’s pregnancies before the clinic opened were the comparator.	ANC Significant increases in %: Women receiving ANC, from 27 (31.8%) before to 49 (57.6%) after (P = 0.004) Women having at least 1 ANC visit with obstetrician, from 23 (27.1%) to 38 (44.7%) (P = 0.003) Women having 3+ ANC visits with nurse, from 21 (24.7%) to 33 (38.8%) (P = 0.011) Non-significant increase in % women registering for care in first trimester, from 3 (3.5%) to 6 (7.1%) (P = 0.230)	Moderate	The intervention recognized that women’s access to care was limited by gender-based restrictions on travel, and established a 3km maximum travel distance for women to reach services. A local Bedouin female liaison worker was used to help women access services. Inadequate childcare provision continued to impact on women’s ability to use services.
Gabrysch et al. 2009	Santillana district, Ayacucho, Peru—Poor, indigenous women	A culturally appropriate model for care at birth was developed with the participation of indigenous communities. Key features included a rope and bench for vertical birthing position, inclusion of family and TBAs during birth, use of the Quechua language, allowing women to wear their own clothes, familial placenta burial, replacement of gynaecological beds with normal beds, and health professionals that were respectful of culture.	Comparison of use of care in the intervention area before and after the intervention. The study used data from a routine monitoring system on births at baseline in 1999 (*n* = 52) and in 2007 (*n* = 83). Qualitative data from an evaluation of the project in 2001 were also reported.	Facility birth At baseline in 1999 (n = 52): 6% births at health centre 37% births with a health professional at home In 2007 (n = 83): 83% births at a health facility 95% births with skilled attendants Most change in the first 2 years after implementation in 2000. No significance tests reported	Weak	The intervention recognized the need to respect traditional practices, including family companions during the birth process. Women had experienced disrespectful interactions with providers, including discrimination, and the intervention had to build trust with the community.
Jan et al. 2004	Western Sydney, Australia Aboriginal women	Daruk Aboriginal Medical Service—a community-controlled health service with a midwifery programme staffed by a team including an Aboriginal health worker. Intervention included: transportation, reduced waiting times, informal childcare, female doctors, and home visits. Cultural awareness sessions were also provided for hospital staff, with intervention acknowledgement that service use is influenced by a range of factors including: low education, low income, high unemployment and women’s reports of racial discrimination by service providers.	Retrospective comparison using antenatal record data for Aboriginal women who gave birth in two relevant hospitals (Nepean and Blacktown): an intervention group who received ANC through the programme and a control group who received regular ANC at the relevant hospitals. 185 of 245 records were accessed for the intervention group, and compared with 105 records from Nepean and a random sample of 90 records from Blacktown. Qualitative and cost data were also reported.	ANC Mean number of antenatal visits: Intervention group (Daruk)=10.5 Control groups=5.5 (P < 0.01) at Nepean and 9.5 (P < 0.2) at Blacktown Mean gestational age at first visit (in weeks): Intervention group (Daruk)=17.2 Control groups=21.2 (P < 0.01) at Nepean and 19.9 (P < 0.02) at Blacktown Attended routine antenatal tests: Intervention group (Daruk)=94% Control groups=71% (P = 0.01) at Nepean and 84% (P = 0.02) at Blacktown	Weak	The intervention recognized that: women had experienced disrespectful interactions with providers, including discrimination; women would not return for services if they perceived a male doctor behaving in a superior way to them; hospital care for Aboriginal women is disempowering; and, there was poor communication by providers with women. Despite the intervention, transport problems persisted (low private transport ownership, inadequate public transport). Inadequate childcare (exacerbated by transport problems) remained an issue, despite informal childcare provision by the intervention. Aboriginal-specific services were considered stigmatizing by some Aboriginal women.
Jewell and Russell 2000	Indiana, USA Racial and ethnic minority women	Minority health coalitions developed projects to increase access to early ANC for minority women through community outreach and eliminating cultural barriers to care. Strategies included using minority professional and paraprofessional staff, social support, advocacy, and referrals for health education and transportation. Intervention staff focused on supporting women to navigate service provision, and to make decisions situated within womens’ cultural beliefs and practices.	Retrospective comparison of birth certificate data of infants born to project mothers and those born to non-project mothers. The intervention group comprised all enrolled women who had a birth during a 2-year project period (*n* = 95). The control group comprised a stratified random sample of mothers in corresponding counties (*n* = 188). Birth certificates were matched on race, age, marital status, and educational attainment.	ANC Mean number of ANC visits: Intervention group=11.5 Control group=9.2 (P = 0.001) ANC in first trimester: Intervention group=73.3% Control group=53.3% (P = 0.01)	Weak	The intervention recognized that minority women’s lower use of early ANC was a function of factors including: provider cultural insensitivity, with some women experiencing disrespectful interactions with providers, including discrimination; and, the important role of informal advice in women’s care-seeking.
Julnes *et al*. 1994	Norfolk, Virginia, USA Latina adolescents at high-risk for poor pregnancy and care outcomes	Norfolk Resource Mothers Program—a community outreach programme that used ‘resource mothers’—lay people often sharing cultural background with the adolescents—to assist with non-medical dimensions of pregnancy and childcare, including getting ANC and acting as a liaison between them and public agencies. The resource mothers were identified as being particularly able to provide empathy for adolescents’’ needs and situations.	Retrospective comparison of women in the programme area who were in the intervention group (*n* = 49) with 1/women who attended a clinic-based, multi-disciplinary programme (*n* = 46); 2/women who had no ANC (*n* = 29). The study used a database constructed from monthly reports of births in the programme area, based on birth certificate information.	ANC More than 6 ANC visits: Intervention group=87.8% Control group(1)=73.9% (P < 0.10) ANC before fourth month of pregnancy: Intervention group=53.1% Control group(1)=32.6% (P < 0.05) Facility birth No significant difference in births in a non-hospital facility	Weak	Intervention staff reported that, above and beyond the maternal health outcomes reported, the intervention also helped establish linkages for service users with other (non-health) agencies, and provided broader social support for the adolescents and their families.
Marsiglia *et al*. 2010	Phoenix, Arizona, USA Latina/Hispanic women	Familias Sanas—an intervention to bridge the cultural gap between Latinas and the health care system. Bilingual, bicultural Prenatal Partners served as cultural brokers, providing ongoing ANC education and support services. They showed participants how to navigate the health system and helped them improve communication with health care providers.	Randomized controlled trial. Eligible participants who attended the Women’s Care Clinic during pregnancy with no prior ANC visits to the clinic for the current pregnancy were randomized to the treatment (*n* = 221) and control (*n* = 219) groups. The control group received care as usual.	Postpartum care Compliance with 6–8 week postpartum visit: Intervention group=73% Control group=51% (χ2=17.88, 1df, P = 0.000) More meetings with the Prenatal Partner was associated with a greater effect	Weak	The intervention acknowledged health-related Latino cultural and spiritual beliefs, and recognized the importance of familial social support for women seeking care.
Nel and Pashen 2003	Remote northern and western Queensland, Australia Aboriginal women	Problems with ANC from Indigenous communities’ perspectives were identified. A prenatal clinic was then established for Indigenous women at an Aboriginal community-controlled health service, managed by a community board and staffed by Indigenous people. Women were seen in a familiar, culturally appropriate environment, initially by Indigenous staff. Transport was provided and families were involved in care.	Comparison of outcome counts before and after the intervention	ANC Pregnant women presenting at the only hospital in the region offering intrapartum care without any ANC decreased from 10 in the 6 months before implementation to 2 in the 6 months after implementation. Attendance at the clinic increased gradually. No significance tests reported.	Weak	The intervention recognized the importance of the role of the extended family in women’s care-seeking behaviours. Dialogue between service providers and communities considered to be key to service use, including local indigenous representatives that identified service shortcomings.
NSW Health 2005	New South Wales (NSW), Australia Aboriginal women across six Area Health Services	NSW Aboriginal Maternal and Infant Health Strategy—community midwife and Aboriginal health worker teams were established to provide targeted, community-based, culturally appropriate services for Aboriginal women in each area. State-wide training was introduced for these staff. Community development programmes were included to varying degrees across areas.	Comparison of women in the intervention group with 1/aboriginal women in relevant local government areas (LGA) before the intervention; 2/aboriginal women in relevant LGA after implementation. Data included programme-specific data on all births for a 2 year period (n = 689) and population-based data from NSW Midwives Data Collection for 1996–2000 (n = 2275) and 2003 (n = 524). Qualitative data were also reported.	ANC First antenatal visit before 20 weeks gestation: Aboriginal women in LGA pre-intervention (1996–2000)=65% Aboriginal women in LGA in 2003=76% (OR 1.7; 95% CI 1.4–2.2; P < 0.001) Women in intervention group (AMIHS) in 2004=78% (OR 1.2; 95% CI 1.01–1.4; P = 0.03)	Weak	The intervention recognized problems of transport services for women to access healthcare, and this was reflected in the location of services in communities where women could seek care close(r) to home. Some community members continued to have low interest in the service provision, related to the bureaucracy (e.g. registration) involved. Although not measured as an outcome, Aboriginal women in the intervention group reported higher levels of respect from staff.
Panaretto *et al*. 2005	Townsville, North Queensland, Australia Aboriginal and Torres Strait Islander women	Mums and Babies program—collaboration with indigenous communities produced an integrated model of antenatal shared care, delivered from the community-controlled Townsville Aboriginal and Islander Health Service. Strategies included using Aboriginal health workers, continuity of care, and a family friendly environment.	Comparison of women attending the Mums and Babies program and a historical control group. Prospective ANC data for women attending Townsville Aboriginal and Islander Health Service (TAIHS) for the intervention (n = 456) were compared with retrospective data for women who attended TAIHS for ANC before the intervention (n = 84). Note: a control group that did not attend TAIHS for ANC were also included, but were not compared on the use of ANC outcomes reported here	ANC ANC visits per pregnancy, median (inter-quartile range): Intervention group=7(4–10) Control group=3(2–6) P < 0.001 Pregnancies with inadequate care (not defined): Intervention group=19.1% Control group=52.4% (P < 0.001) Weeks of gestation at first visit, median (inter-quartile range): Intervention group=12(8–20) Control group=14(7–22) Not significant But significant for Townsville-based only Pregnancies with late first visit (not defined): Intervention group=11.0% Control group=17.9% (P < 0.05)	Moderate	Authors conclude that longer term success of the intervention is likely to be dependent on quality of community-service provider relationships, and the presence of individuals to champion the intervention.
Panaretto *et al*. 2007	Townsville, North Queensland, Australia Aboriginal and Torres Strait Islander women	Mums and Babies program—see Panaretto *et al*. 2005 for details	Comparison of women attending the Mums and Babies program and a historical control group. Prospective ANC data for women attending Townsville Aboriginal and Islander Health Service (TAIHS) for the intervention (*n* = 781) were compared with retrospective data for women who attended TAIHS for ANC before the intervention (*n* = 84).Note: the intervention group includes the *n* = 456 from Panaretto *et al*. (2005) and later intervention participants	ANC ANC visits per pregnancy, median (inter-quartile range): Intervention group=6(4–10) Control group=3(2–6) P < 0.001 Weeks of gestation at first visit, median (inter-quartile range): Intervention group=13(8–20) Control group=14(7–22) Not significant But significant for Townsville-based only Pregnancies with inadequate care (not defined), (Townsville-based women only) Intervention group = 16.7% Control group = 52.4% (P < 0.001) Pregnancies with late first visit (not defined): Intervention group=11.5% Control group=17.9% (P = 0.004)	Weak	Continuous (long-term) project evaluation considered to have led to a culture of change among service providers, including greater staff education and better staff retention.
No effect on care utilization						
Mason 1990	Leicestershire, UK Asian women	The Asian Mother and Baby Campaign—eight linkworkers were distributed equally between the hospital and community setting. The linkworkers—women aged 20–45 and fluent in English and an Asian language—worked alongside health professionals as ‘facilitators’ and ‘interpreters’, whilst also fulfilling an educational role.	Comparison of outcomes between three groups of women who saw a linkworker and a control group that did not. Women from selected general practices with higher than average perinatal risk were interviewed using a medical/health knowledge questionnaire: 1/at booking for ANC; 2/after birth; 3/at the postnatal visit. The study enrolled a total of 485 Asian women.	ANC No difference in mean number of ANC visits No significant difference in the proportion of women receiving ANC by 12 weeks of gestation	Weak	There were problems in recruitment of female doctors, which Asian women in this setting preferred. Communication problems persisted when the cultural broker could not be present at an appointment.
McQuestion and Velazquez 2006	12 of 25 departmentos across Peru Communities in high-risk distritos	Proyeto 2000—a project to make emergency obstetric care (EmOC) services culturally acceptable, woman-friendly, and high-quality. Local birthing practices were incorporated into clinical protocols (specific features were not described). Qualitative data collected on mothers’ perceptions and preferences also informed a multimedia Safe Motherhood campaign; TBAs were trained; and facility staff engaged new community health committees.	An endline survey with 5335 mothers in the catchment areas of 29 treatment and 29 matched control EmOC facilities. The probability of birth at the nearest public EmOC facility was modelled, conditional on whether the mother’s area participated in the programme, among other factors.	Facility birth Living in a Proyecto 2000 area had no significant effect on institutional births (coefficient 0.79; SE 0.25).	Weak	The intervention was designed to acknowledge a range of problems, including: widespread discrimination and mistreatment of women by health workers; low levels of understanding of Spanish among clients, combined with providers that only spoke Spanish; and, services that did not respect women’s privacy or their cultural values and norms. Despite the intervention including an extension to the Maternal and Child Health Insurance Program, fees remained a barrier to uptake of services.
Parsons and Day 1992	Hackney, East London, UK Non-English speaking women	The Multi-Ethnic Women’s Health Project—a health advocacy programme introduced at a hospital to meet the needs of non-English speaking women. Health advocates interpreted and mediated between service users and professionals to ensure an informed choice of health care.	Retrospective comparison of women who received the intervention with geographical and historical controls. Clinical data for 1000 non-English-speaking women who had a birth at Mothers’ hospital and received the intervention were compared with: 1/1000 non-English-speaking women had a birth at Mothers’ hospital before the intervention; and 2/two similar groups from a reference hospital (Whipps Cross) that did not receive the intervention.	ANC Weeks of gestation at first booking: Mothers’ Hospital=19.5% (1979), 18.8% (1986); Whipps Cross=17.7% (1979), 16.8% (1986) Women booked significantly earlier at both hospitals in 1979 and in the control hospital in 1986. But no evidence that change was different between the intervention and control hospitals Non-attendance of antenatal appointments: Mothers’ Hospital=7.2% (1979), 8.4% (1986); Whipps Cross=4.5% (1979), 4.6% (1986) Non-attendance rose slightly for both hospitals, more at the Mothers’ Hospital than at Whipps Cross (difference not significant).	Moderate	The authors emphasize that the autonomy of the health advocates from the healthcare providers was key to the intervention, although no significant effect on care use was found. The authors suggest that the intervention had other, wide-reaching impacts including: changes to hospital food; a reduction in racist behavior by staff; and, changes to clinical protocols. A major constraining factor was the supply of female doctors to meet patient preferences.
Thompson *et al*. 1998	Rural Oregon, USA Low-income, rural Mexican-American women at risk of poor pregnancy outcomes	The Rural Oregon Minority Prenatal Program—a programme that blended culturally appropriate care with outreach, nursing case management, and home visitation to facilitate access to ANC and community services. A bilingual, bicultural outreach worker served as a cultural broker. She ensured continuity of care, reduced social isolation, and provided translation, advocacy and transportation.	Retrospective comparison of women who received the intervention (*n* = 100) with demographically similar women who gave birth in the study county in the 2 years prior to the intervention (*n* = 100). The study used birth certificate and medical record data.	ANC No statistically significant effect on adequacy of initiation of ANC or adequacy of the total number of ANC visits, using the Adequacy of Prenatal Care Utilization index (Kotelchuk, 1994).	Moderate	The authors identify structural issues that might have reduced the intervention success, including: financial barriers to service use for poor women; transport problems; inadequate childcare (exacerbated by transport problems); and, provider attitudes and lack of familiarity with this population. It is possible that outreach services might have been seen as a substitute for ANC, rather than supplementary.
Negative effect on care utilization						
Kildea *et al*. 2012	Brisbane, Australia Aboriginal and Torres Strait Islander women	Murri clinic—an antenatal clinic established in a tertiary hospital to provide services to Aboriginal and Torres Strait Islander women. Services included an Indigenous midwife and Indigenous liaison officers, who helped families feel welcome, provided support for women in rural and remote areas, and served as cultural brokers.	Retrospective comparison of women who attended the hospital’s Murri Clinic for ANC (n = 367) and Indigenous women who received standard care in the same hospital over the same period (n = 414). The study used routinely collected clinical data from obstetric databases. Qualitative and survey data were also reported.	ANC Number of ANC visits at Murri or hospital: 2–4=15.8% (Murri), 11.0% (standard care) (P = 0.007) 5–7=23.6% (Murri), 19.7 (standard care) 8 + =28.1 (Murri), 37.7 (standard care)	Weak	Male partners continued to feel uncomfortable with accompanying women, which affected women’s use.Transport problems persisted (low private transport ownership, inadequate public transport).Inadequate communication between intervention services and mainstream services led to some duplication of provision.

Inevitably, interventions differed according to the population targeted and cultural factors affecting use of services. Interventions varied in comprehensiveness; some studies described adaptations to an otherwise standard model of care (e.g. Julnes *et al.* 1994; Jewell and Russell 2000), whereas others described comprehensive interventions to provide distinct, culturally tailored maternity care services (e.g.: Bilenko *et al.* 2007; Gabrysch *et al.* 2009). Correspondingly, some interventions focused on a single strategy (e.g.: Mason 1990), whereas others adopted multiple strategies (e.g. Thompson *et al.* 1998; Nel and Pashen 2003).

Strategies focusing on service providers were most common. In particular, cultural-appropriateness was sought via the use of staff who shared cultural and/or linguistic backgrounds with service users. These staff ranged from health professionals to intermediaries who bridged the cultural or linguistic gap between health professionals and service users. A smaller number of studies described training existing staff to improve cultural awareness. Community participation was a strategy explicitly described in multiple studies, albeit to varying extents. Approaches ranged from consulting communities in the design of intervention strategies to providing community-controlled health services. Further strategies included incorporating local birthing practices into service provision; adapting physical and social service settings for familiarity and comfort; and adapting educational materials. Since cultural factors interact with social and economic factors to affect these populations’ access to maternity care services, they were frequently addressed jointly in interventions. For example, several interventions incorporated transportation, childcare and/or outreach care.

### Outcome measures

Among relevant outcomes, most studies focused on ANC with a skilled attendant, but measures were wide-ranging. Number of ANC visits was the most common measure (*n* = 7). However, any ANC (Bilenko *et al.* 2007); three or more visits (Bilenko *et al.* 2007); six or more visits (Julnes *et al.* 1994) and non-attendance of appointments (Parsons and Day 1992) were also reported in some papers. Further, Thompson *et al.* (1998) included as a measure the adequacy of the total number of ANC visits, based on the Adequacy of Prenatal Care Utilization index (Kotelchuk 1994), in which the adequate number is dependent on the timing of initiation of care. Timing of initiation of ANC was also a commonly reported outcome, with typical measures including first visit before a specified stage of pregnancy (*n* = 5) or gestational age at first visit (*n* = 4). Three papers—including the two from Peru—reported outcomes for care at birth, including birth with a skilled attendant (*n* = 1) and birth at a health facility (*n* = 3). Only one study reported outcomes related to postpartum care services (Marsiglia *et al.* 2010).

Only four studies mentioned outcomes related to cost and sustainability (Julnes *et al.* 1994; Jan *et al.* 2004; Gabrysch *et al.* 2009; Marsiglia *et al.* 2010). Details were rather limited in two studies with one paper praising the programme’s ‘relatively low cost’ (Julnes *et al.* 1994) and another paper noting that trained lay workers could easily replicate the ‘very cost-effective’ intervention (Marsiglia *et al.* 2010).

### Study design and quality

Table 2 presents a summary of study designs and quality. Only one study used an experimental design (Marsiglia *et al.* 2010). All others used various forms of observational designs. By far the most common type of design was a comparison of outcomes between women who received the intervention and controls, largely using retrospective data from birth certificates or service/clinical records. Controls in these studies included target populations before the intervention (Parsons and Day 1992; Thompson *et al.* 1998; NSW Health 2005; Panaretto *et al.* 2005, 2007) or contemporary controls matched on certain characteristics, who received standard services (Julnes *et al.* 1994; Jewell and Russell 2000; Jan *et al.* 2004; Kildea *et al.* 2012). The latter were essentially types of retrospective cohort studies. Overall the quality of evidence for determining impact was weak. Four studies were assessed to be of moderate quality and all others of weak quality.

### Intervention impact

Table 2 presents a summary of intervention effects on uptake of skilled maternity care services. Eight of 12 studies reported improvements in use and/or timing of initiation of ANC (Julnes *et al.* 1994; Jewell and Russell 2000; Nel and Pashen 2003; Jan *et al.* 2004; NSW Health 2005; Bilenko *et al.* 2007; Panaretto *et al.* 2005, 2007). One of three studies reported increases in birth at a health facility (Julnes *et al.* 1994; McQuestion and Velasquez 2006; Gabrysch *et al.* 2009). The one study that considered postpartum care reported a positive effect (Marsiglia *et al.* 2010). Two studies that reported improvements did not report significance tests (Nel and Pashen 2003; Gabrysch *et al.* 2009). Only two studies that reported positive effects were assessed to be of moderate quality (Panaretto *et al.* 2005; Marsiglia *et al.* 2010).

Studies reporting positive effects encompassed four of five interventions with Aboriginal populations in Australia, all of which reported greater use and/or earlier initiation of ANC in the intervention group (Nel and Pashen 2003; Jan *et al.* 2004; NSW Health 2005; Panaretto *et al.* 2005, 2007). These interventions were often delivered through separate, tailored services, and combined multiple strategies. Core strategies included Indigenous staff; community control and/or participation; a community setting and/or outreach; and a culturally friendly setting and service.

Three of four interventions in the USA also reported positive effects: two on use and timing of ANC and the other on use of postpartum care (Julnes *et al.* 1994; Jewell and Russell 2000; Marsiglia *et al.* 2010). All three used lay or para-professional staff who shared cultural characteristics and/or language with the target population and who provided a range of educational and support services within an outreach model. In various ways, they acted as a link between service providers and users. An intervention in Israel to establish a local maternal and child health clinic in a desert area, staffed by an Arabic-speaking Bedouin public health nurse increased the percentage of women receiving ANC but did not lead to earlier initiation of care (Bilenko *et al.* 2007). Finally, a new model for care at birth in Peru saw a substantial increase in facility birth (Gabrysch *et al.* 2009).

Four studies reported no significant effects on care utilization (Mason 1990; Parsons and Day 1992; Thompson *et al.* 1998; McQuestion and Velasquez 2006), of which two were assessed to be of moderate quality (Parsons and Day 1992; Thompson *et al.* 1998). One study in Australia also reported a negative effect on number of ANC visits, albeit positive effects on other maternal and newborn outcomes not addressed in this review (Kildea *et al.* 2012). Two early interventions from the UK that showed no impact on ANC outcomes used link-workers (Mason 1990) or health advocates (Parsons and Day 1992) to interpret and mediate between service providers and users. A programme in the USA that used a bilingual, bicultural outreach worker showed no effect on adequacy of ANC despite sharing characteristics with other interventions from the USA that demonstrated positive effects (Thompson *et al.* 1998). An intervention in Peru that sought to make emergency obstetric care services culturally acceptable showed no effect on facility birth, but little specific detail was provided on the service adaptations that took place (McQuestion and Velasquez 2006). Across these studies, authors highlighted study design issues and low statistical power as potential explanations for finding no impact. They also highlighted that interventions may not have surmounted all factors affecting uptake, such as poor communication; mistrust and negative attitudes between service providers and users (Mason 1990; Thompson *et al.* 1998; McQuestion and Velasquez 2006); poor communication between hospital and community-based providers (Kildea *et al.* 2012); and financial and transport barriers (Thompson *et al.* 1998).

## Discussion

The studies reviewed suggest that interventions to provide culturally appropriate maternity care services largely have positive effects on use of skilled maternity care. Limitations of the evidence currently available, however, prevent definitive conclusions on modes of delivery of similar interventions and on what works during pregnancy, for birth or in the postpartum period with different types of cultural groups in different contexts.

A substantial number of interventions has been implemented across world regions to address cultural factors affecting use of maternity care services (Coast *et al.* 2014), but few have been evaluated with designs that can demonstrate their impact on use of services. Evidence in this review is clustered within a small number of countries. Evidence from LMICs is notably lacking. Although we found 54 items from LMICs in the precursory mapping (Coast *et al.* 2014), only two of the 14 intervention studies that met inclusion criteria for this review were conducted in LMICs. In particular, despite much recent work in Latin America to develop intercultural approaches to improve Indigenous populations’ access to maternity care services, we identified only two studies with designs to measure impact. While studies of intercultural approaches have provided useful lessons for their further development (Nurena 2009; Tucker *et al.* 2013; van Dijk *et al.* 2013), future studies should also use designs that allow evaluation of their impact. Even studies on high-income countries (HICs) were clustered predominantly in Australia and the USA, with a focus on specific populations in these countries. Given the urgency of relatively poor maternal and newborn health outcomes among many minority and migrant groups in HICs (Karl-Trummer *et al.* 2006; Wasserman *et al.* 2007), the current lack of intervention studies deserves urgent attention.

Because most of the included studies focus on ANC outcomes, evidence of impact is particularly limited for care at birth and after birth. It is unclear why the focus is largely on pregnancy. There is also no measure or discussion of whether the increase in ANC use leads to increased use of skilled care at birth or if women are satisfied with the service. The limited focus on postpartum care is noteworthy given that low uptake of postpartum care is a widespread problem. In sum, the scope of the evidence on impact across the continuum of maternity care is currently limited.

The EPHPP tool for quantitative studies looks at different domains to assess the quality of a study, including selection bias, study design, confounders, blinding, data collection methods, withdrawals and drop-outs. Most studies were determined to be of low quality, and we are aware of the potential biases arising from the study designs. In order to determine the effectiveness of interventions in terms of their contribution to achieving the desired outcomes, future studies should seek to employ more robust designs and reduce biases through randomization and/or more robust control of confounding factors. Several evaluations were done retrospectively, taking advantage of local databases, clinical records and registers. However, designing evaluations into the intervention from the outset provides more control in achieving optimal study designs.

Interventions to provide culturally appropriate maternity services are by nature context-specific. Yet, there are certainly elements that would benefit from better definition and standardization so that they contribute to a body of evidence rather than a disparate collection of studies. This would help to determine the effectiveness of these interventions. It seems that bodies of evidence are developing for interventions with specific populations in some countries or world regions, notably Australia and parts of Latin America (Wasserman *et al.* 2007; Castro 2012; Kildea and Van Wagner 2012; Jongen *et al.* 2014). Yet, intervention studies from many other countries that have documented the impact of cultural factors on use of care are disconnected. This is perhaps unsurprising given the challenging process of collating papers on interventions that have addressed cultural factors in maternity care services (Coast *et al.* 2014). This review, as well as other recent reviews for interventions in specific contexts (Kildea and Van Wagner 2012; Jongen *et al.* 2014), provides a starting point for such efforts. Researchers should be clear on the research question, determine an optimal study design, seek to define the intervention and its context carefully, standardize outcome measurements while allowing local contextualization, and document important process issues (WHO 2015b).

Limitations of this review are acknowledged. In brief, culture is a complex, elusive concept that is inconsistently defined in the literature, and is susceptible to over-generalization. This issue is compounded by poor reporting, where authors do not always describe carefully what they seek to address or how they address it (Coast *et al.* 2014). Both issues posed challenges during the screening process. Second, publication bias is a possibility. Third, it is possible that we missed some relevant literature, most likely grey or non-English-language literature, despite extensive efforts to uncover such evidence through expert recommendations. Literature reviews of interventions in Australia (Kildea and Van Wagner 2012) indicate that evaluations of such interventions may sometimes be documented only within in-service or programme reports that are less likely to be uncovered by database searches or even expert suggestions. Our global focus meant that we were unable to follow-up on such avenues in each country to the extent possible in more context-focused reviews, such as Kildea *et al.* (2012). Further, while we included eligible French and Spanish items that we found, and employed a variety of search methods, the possibility that we missed some relevant non-English literature is higher. Although we included items in Spanish, it is possible that some relevant work from Latin America was not uncovered because of biases in database and online inclusion.

In sum, the combination of overall weak evidence for determining impact; diverse interventions with multiple measures; and heterogeneous but limited coverage of populations and settings constrains our ability to conclude what interventions—or elements of them—work (or not). Nevertheless, this review reveals that most studies of interventions to provide culturally appropriate maternity care services have demonstrated positive effects. Moreover, they lend tentative support to some intervention elements that have been highlighted fairly consistently within the current evidence base. In particular, the review and the broader literature confirm that cultural factors are often not the sole factor affecting minority populations’ use of maternity care services. Broader social, economic, geographical and political factors interacted with cultural factors to affect targeted populations’ access to services in included studies (Bohren *et al.* 2014). These factors should be considered and, where appropriate or possible, addressed within and/or alongside interventions to provide culturally appropriate services. As Jan *et al.* (2004) stated, ‘With a service of this nature, it is often not possible to provide care in isolation from the social problems in which many of the women live’. The depth of context-specific information included in the fifteen studies was highly variable, but across a range of settings, lack of transport (Israel, Australia, USA), financing (USA, Peru) and need for support for childcare (Israel, Australia) are highlighted by authors to explain additional barriers to use of skilled maternity care. Language, and the implications for woman-provider communication, was an almost universal issue across the studies, with the exception of studies from Australia.

The highly constrained evidence base on the effectiveness of interventions in LMICs, particularly outside of Latin America, reflects that whilst issues of culture are being recognized by programmes and researchers as being important, interventions that explicitly incorporate issues of culture are not being evaluated. We surmise that in settings where minimum standards of skilled maternity care are unavailable, the evidence base emphasis continues to focus on the supply side of technical care provision. Demand-side interventions tend to focus on more easily measurable socio-demographic characteristics such as education, age or wealth. Without evidence that demonstrates the centrality of culture to women’s demand for, and use of skilled maternity care, the policy and provision gap will persist.

Finally, it is notable that principles espoused in these studies of interventions to make services culturally appropriate share many characteristics with recent shifts in maternal health policy discourse more generally. Woman- and family friendly environments, respect for culture, and emphasis on respectful interpersonal interaction overlap in many ways with the principles of respectful and humanized care that should be available universally to all women (Langer *et al.* 2013; Windau-Melmer 2013).
